# Calcific Tendinitis: A Pain‐Oriented and Site‐Specific Narrative Review

**DOI:** 10.1155/prm/9952967

**Published:** 2026-07-11

**Authors:** Jiaqi Li, Xiaodi Zou, Hui Lu, Tao Jiang

**Affiliations:** ^1^ Department of Orthopedics, The Second Affiliated Hospital of Zhejiang Chinese Medical University, Hangzhou, 310005, Zhejiang, China, z2hospital.com; ^2^ Department of Orthopedics, The First Affiliated Hospital, Zhejiang University, #79 Qingchun Road, Hangzhou, 310003, Zhejiang, China, zju.edu.cn

**Keywords:** bursitis, extracorporeal shockwave therapy, hydroxyapatite deposition disease, shoulder pain, tendinopathy, ultrasonography

## Abstract

Calcific tendinitis is a common musculoskeletal disorder characterized by deposition of calcium hydroxyapatite within or around tendon tissue. Although it has traditionally been regarded as a structural tendon pathology, growing clinical evidence suggests that symptom severity, particularly pain, is more closely related to inflammatory activity and disease stage than to the size or precise anatomical location of calcific deposits. In routine practice, patients most often seek medical attention during sudden and intense pain episodes, typically occurring in the resorptive phase. Current diagnostic frameworks and treatment strategies are largely derived from studies on rotator cuff calcific tendinitis. In contrast, calcific tendinitis involving nonrotator cuff sites remains under‐recognized and is frequently misdiagnosed. These atypical presentations commonly manifest with acute inflammatory pain that mimics infection, fracture, or inflammatory arthropathy, often resulting in diagnostic confusion and unnecessary interventions. This narrative review adopts a pain‐oriented, site‐specific framework to examine calcific tendinitis, with emphasis on nonrotator cuff involvement. By integrating pain presentation, disease stage, and targeted imaging, we highlight a symptom‐driven approach to diagnosis and management. Conservative care with site‐specific rehabilitation remains fundamental, while invasive treatments should be reserved for refractory pain.

## 1. Introduction

Calcific tendinitis is a frequently encountered musculoskeletal disorder defined by the deposition of calcium hydroxyapatite crystals within or adjacent to tendon tissue [1, 2]. For many years, it was viewed primarily as a structural abnormality, with clinical attention centered on the presence, size, and anatomical location of calcific deposits [3]. This structure‐focused perspective, however, often fails to explain a familiar clinical paradox: patients with substantial calcification may remain minimally symptomatic, whereas others experience sudden, severe pain despite relatively modest imaging findings [3, 4].

From a pain‐management standpoint, patients rarely present because of chronic calcification itself [5]. Instead, medical attention is typically sought during abrupt pain exacerbations that are intense, disabling, and disproportionate to structural changes [4]. Accumulating clinical and experimental evidence indicates that these pain flares are closely linked to inflammatory activity during the resorptive phase of the disease rather than to progressive tendon degeneration or increasing calcific burden [3–5]. As a result, calcific tendinitis is increasingly recognized as a dynamic, stage‐dependent, and pain‐dominant condition [6].

The shoulder rotator cuff remains the most extensively studied site of calcific tendinitis and has shaped widely accepted pathogenetic models, imaging classifications, and treatment strategies [1, 7–9]. The classic stage‐based framework of calcific tendinopathy outlines a sequence of precalcific changes, a calcific phase with formative, resting, and resorptive subphases, and an eventual postcalcific remodeling stage involving tendon repair and granulation tissue replacement. This reactive calcification cycle was originally described by Uhthoff and Loehr, and subsequent imaging and histopathological studies have detailed the fibrocartilaginous metaplasia of tenocytes, phased calcium deposition, intense inflammatory resorption, and tendon remodeling in the postcalcific phase [1, 3, 10]. Notably, pain intensity typically peaks during the resorptive stage of calcific tendinopathy and often manifests as sudden, severe shoulder pain despite minimal changes on static imaging, underscoring the limited value of conventional radiographs and other static imaging findings in predicting symptom severity or clinical course [8, 10].

By contrast, calcific tendinitis involving nonrotator cuff sites has received far less attention. These presentations, affecting regions such as the hip, lower limb, or distal upper extremity, are clinically important precisely because they often present with abrupt, severe pain accompanied by prominent inflammatory features [11]. In routine practice, such cases frequently mimic infection, fracture, or inflammatory joint disease, placing patients at risk of misdiagnosis, unnecessary antibiotic treatment, or invasive procedures [12, 13].

**FIGURE 1:**
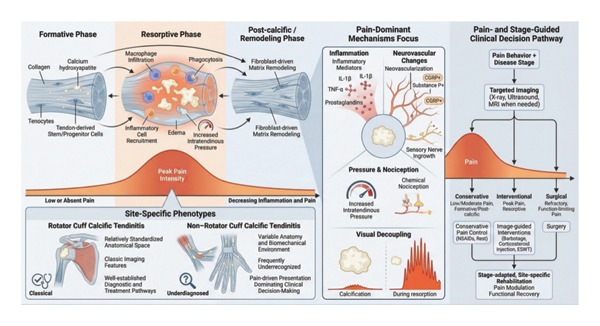
A pain‐oriented and stage‐adapted conceptual framework for calcific tendinitis. Calcific tendinitis is best conceptualized as a dynamic, stage‐dependent, and pain‐dominant condition rather than a purely structural tendon disorder. This framework illustrates how cellularly mediated calcification, inflammatory resorption, and neurovascular changes interact across disease stages to shape pain presentation. Pain severity typically peaks during the resorptive phase and correlates more closely with inflammatory activity than with calcification size or anatomical location. The model further highlights important site‐specific differences, contrasting the relatively standardized presentation of rotator cuff calcific tendinitis with the frequently under‐recognized and pain‐driven manifestations at nonrotator cuff sites. Diagnostic and therapeutic decision‐making is therefore guided primarily by pain behavior and disease stage, integrating targeted imaging, stepwise treatment escalation, and stage‐adapted, site‐specific rehabilitation to optimize pain control and functional recovery while avoiding unnecessary interventions. This figure is an original conceptual framework created by the authors of this manuscript.

Accordingly, this review is intentionally designed as a narrative synthesis rather than a systematic analysis. Based on clinical experience and available evidence, we adopt a pain‐oriented and site‐specific perspective, with particular emphasis on representative nonrotator cuff presentations. The selected anatomical sites were chosen because they are among the more clinically recognized nonrotator cuff forms, frequently present with acute inflammatory pain and diagnostic uncertainty, and have relatively available imaging and clinical literature to support site‐specific discussion. Accordingly, this review is not intended to provide an exhaustive overview of all reported anatomical locations, but rather to highlight clinically relevant patterns that best illustrate pain‐oriented diagnostic and therapeutic challenges. By integrating current knowledge on pathogenesis, diagnostic strategies, treatment options, and rehabilitation principles, we aim to support a clinically pragmatic approach that aligns management decisions with pain behavior and disease stage rather than with calcification morphology alone. The conceptual relationship between disease stage, inflammatory activity, pain behavior, and site‐specific clinical manifestations is summarized in Figure 1.

## 2. Pathogenesis

### 2.1. Evolution of Pathogenetic Concepts

Calcific tendinitis was long regarded as a passive degenerative process, attributed to tendon wear, local ischemia, or tissue necrosis, ultimately leading to dystrophic calcification, although more modern studies have emphasized active, cell‐mediated mechanisms rather than simple degeneration [3, 10, 14]. While intuitively appealing, this explanation fails to account for several consistent clinical observations. Calcific deposits frequently develop in structurally preserved tendons, the condition predominantly affects middle‐aged individuals rather than the elderly, and spontaneous resolution is common [3, 14]. These features are difficult to reconcile with a purely degenerative model. Histological studies have further challenged this traditional view by demonstrating calcific deposits embedded within viable, well‐vascularized tendon tissue rather than necrotic areas [3]. Such findings have driven a conceptual shift toward understanding calcific tendinitis as an actively regulated and potentially reversible biological process [6, 15]. From a clinical standpoint, this shift is crucial, as it helps explain why pain behavior and disease course often diverge from static structural imaging findings.

### 2.2. Tenocyte Phenotypic Transformation

Current evidence supports the notion that calcific tendinitis arises from aberrant cellular behavior within tendon tissue. Under conditions such as local hypoxia, altered mechanical loading, or metabolic stress, resident tenocytes and tendon progenitor cells may undergo fibrocartilaginous metaplasia and adopt hypertrophic chondrocyte‐like phenotypes [1, 16]. These transformed cells exhibit increased matrix proteoglycan characteristics and express mineralization regulators, notably tissue‐nonspecific alkaline phosphatase (TNAP), creating a permissive microenvironment for hydroxyapatite crystal deposition and pathological mineralization within the tendon [17, 18].

In addition to mature tenocytes, resident tendon‐derived progenitor cells (TSPCs/TDPCs) appear to contribute to the pathogenesis of calcific tendinitis [19]. These cells exhibit multipotent differentiation potential and, under altered microenvironmental cues such as mechanical overload, hypoxia, or inflammation, may undergo dysregulated chondrogenic or osteogenic differentiation rather than tenogenic maturation, contributing to abnormal extracellular matrix remodeling and mineral deposition in tendon tissue [20, 21]. This supports a model in which calcific tendinitis reflects a maladaptive, cell‐mediated response to microenvironmental stress rather than a passive consequence of tissue breakdown [15].

### 2.3. Inflammatory and Pain‐Related Mechanisms

Inflammation plays a central role in symptom generation in calcific tendinitis, particularly during the resorptive phase [10]. This stage is characterized by neovascularization and infiltration of macrophages and multinucleated giant cells actively involved in calcium resorption [6]. Clinically, this inflammatory resorptive phase coincides with abrupt and often severe pain exacerbations, which are typically disproportionate to imaging findings and reflect the underlying inflammatory and vascular response.

Multiple mechanisms are likely involved in pain generation during the resorptive phase of calcific tendinitis. Chemical irritation and swelling from calcium deposits can lead to tissue edema and increased intratendinous pressure, mechanically sensitizing local nociceptors and contributing to severe acute pain [10, 22]. Extrusion of calcific material into adjacent bursae or soft tissues can amplify local inflammation and impingement‐related discomfort [1]. Histologically, the resorptive phase is marked by neovascularization and infiltration of inflammatory cells (macrophages, mast cells) that release pro‐inflammatory mediators. In parallel, there is evidence of neoinnervation and upregulation of pain signaling pathways in affected tendons, further enhancing peripheral nociceptive signaling [23, 24]. Together, these processes support the view that pain in calcific tendinitis is primarily a transient inflammatory overload response rather than a direct indicator of gross structural tendon damage.

### 2.4. Stage‐Based Disease Model

The stage‐based model of calcific tendinitis remains a useful framework for linking biological processes to clinical behavior. Historically described as a transition through precalcific, calcific (comprising formative, resting, and resorptive phases), and postcalcific remodeling stages, this model maps to distinct cellular and morphological events—from fibrocartilaginous metaplasia and hydroxyapatite deposition to cell‐mediated resorption and tissue remodeling [7, 10, 14, 22]. Importantly, these stages are not strictly sequential in all cases, and overlapping features (e.g., simultaneous resorption and early repair) are frequently observed in clinical and imaging studies, underscoring the dynamic, nonlinear nature of the disease process [10].

From a pain‐oriented perspective, the resorptive phase of calcific tendinitis is of particular clinical relevance, as it is typically associated with intense inflammation and severe acute pain, whereas the precalcific and formative/resting phases are often asymptomatic or only mildly symptomatic [7, 10, 25]. This stage‐dependent symptom variability highlights that crisp imaging morphology alone does not fully capture a patient’s clinical presentation; instead, integrating pain symptoms with imaging findings is essential to guide appropriate management decisions.

### 2.5. Mechanical and Microenvironmental Influences

Mechanical loading patterns and the local tendon microenvironment appear to modulate both disease initiation and symptom expression [26]. Calcific deposits preferentially develop in regions of relative hypovascularity subjected to repetitive mechanical stress, suggesting a contributory role for local hypoxia and microtrauma [27]. These factors may act as triggers for cellular phenotypic change and subsequent mineralization [1, 3, 4].

During inflammatory phases, angiogenesis and nerve ingrowth further alter the tendon microenvironment, amplifying nociceptive signaling and symptom severity [3, 28]. The interaction between mechanical factors, cellular responses, and pain pathways underscores calcific tendinitis as a dynamic condition shaped by converging biological and biomechanical influences rather than by a single pathogenic mechanism [29].

## 3. Site‐Specific Manifestations of Calcific Tendinitis

### 3.1. Rotator Cuff Calcific Tendinitis

Rotator cuff calcific tendinitis represents the most extensively studied and clinically characterized form of calcific tendinitis and serves as the basis for current diagnostic and therapeutic strategies [3, 28]. The condition most commonly affects the supraspinatus tendon, particularly within its hypovascular “critical zone,” and demonstrates a well‐defined stage‐dependent biological behavior [30, 31]. Imaging‐based classification systems using radiography and ultrasonography allow reliable assessment of deposit morphology, consistency, and disease phase, thereby facilitating stage‐oriented clinical decision‐making [32, 33].

Several radiological classification systems are routinely applied in clinical practice to characterize calcific deposits and provide indirect insight into disease stage. On plain radiographs, the Gärtner and Heyer classification remains the most widely used, distinguishing type I (well‐defined, dense deposits), type II (heterogeneous deposits with partially defined margins), and type III (ill‐defined or translucent deposits). These categories broadly reflect the transition from the formative/resting phases to the resorptive phase [7, 34]. Ultrasound‐based classifications offer additional, and often more clinically relevant, information by capturing deposit consistency and biological activity. Systems such as the Sconfienza classification describe calcifications as hard, fragmented, nodular, or fluid‐like, while Doppler signal and posterior acoustic shadowing further assist in identifying inflammatory activity and resorptive changes [7, 35]. In our experience, these sonographic features tend to align more closely with symptom severity than static radiographic appearance alone. Taken together, radiographic and ultrasonographic classifications should not be interpreted in isolation. Rather, they are best integrated with the clinical presentation, as they provide complementary perspectives on both the structural morphology and the underlying biological activity of calcific deposits.

Clinical symptoms, especially pain intensity, correlate more strongly with the biological stage of the disease than with the absolute size of calcific deposits [3, 28]. Patients are frequently asymptomatic or mildly symptomatic during the formative and resting phases, whereas the resorptive phase is characterized by acute inflammation, neovascularization, and marked pain exacerbation that may be disproportionate to structural findings [10, 14]. This stage‐dependent pain pattern underscores the importance of interpreting imaging findings in conjunction with clinical presentation rather than relying solely on calcification dimensions.

Importantly, within the framework of the Uhthoff cycle, calcific deposits should not be viewed as static entities but as dynamic structures capable of fragmentation and migration, particularly during the resorptive phase. At this stage, the deposits become softer and more hydrated, enabling them to disperse through intratendinous cleavage planes under mechanical forces such as compression and tension [36]. These fragmented calcium deposits may extend beyond their original intratendinous location and migrate into adjacent anatomical compartments. Reported migration patterns include intratendinous dispersion, sub‐bursal or intrabursal extension, intramuscular infiltration, and, less commonly, intraosseous or interfascial spread [37, 38]. From a clinical standpoint, this migratory behavior is highly relevant, as it can substantially modify symptom presentation. For example, extrusion of calcific material into the subacromial‐subdeltoid bursa may provoke acute microcrystalline bursitis, resulting in severe inflammatory pain that can mimic infection or inflammatory arthropathy [39]. Similarly, intramuscular or interfascial migration may lead to atypical pain patterns, thereby increasing the risk of diagnostic uncertainty. These migration patterns also carry important therapeutic implications. Bursal involvement may favor ultrasound‐guided bursal injection, whereas intratendinous soft deposits in the resorptive phase are generally more amenable to ultrasound‐guided needling or barbotage [33]. In this context, recognizing the dynamic and migratory nature of calcific deposits is essential for guiding both diagnosis and stage‐specific, targeted intervention.

From a management perspective, rotator cuff calcific tendinitis has benefited from relatively standardized, stage‐adapted treatment algorithms. Conservative therapy remains the first‐line approach for most patients, while interventions such as extracorporeal shockwave therapy (ESWT) and ultrasound‐guided needling are preferentially considered in persistent, painful cases, particularly during or approaching the resorptive phase [34, 40, 41]. Rehabilitation protocols emphasize pain control and maintenance of shoulder mobility in acute stages, followed by progressive strengthening and biomechanical optimization during recovery [9]. Owing to this relatively predictable natural history and evidence‐based management framework, rotator cuff calcific tendinitis provides a reference model against which less common, nonrotator cuff forms can be compared.

### 3.2. Nonrotator Cuff Calcific Tendinitis

Nonrotator cuff calcific tendinitis encompasses a heterogeneous and frequently under‐recognized spectrum of conditions characterized by hydroxyapatite deposition in tendons outside the shoulder rotator cuff. Although less prevalent than rotator cuff involvement, these entities are clinically significant because they often present with abrupt, severe pain that is disproportionate to structural findings on imaging and are associated with a high risk of misdiagnosis [4, 42]. In contrast to rotator cuff disease—where well‐established imaging classifications and treatment algorithms exist—nonrotator cuff calcific tendinitis lacks standardized diagnostic pathways, contributing to delayed recognition and inappropriate management [14, 43].

A unifying feature across nonrotator cuff sites is the predominance of acute inflammatory presentations during the resorptive phase [3]. At this stage, intense local inflammation, soft‐tissue edema, and occasionally reactive laboratory abnormalities may closely mimic infection, fracture, crystal arthropathy, or inflammatory rheumatic disease [42]. As a result, patients are frequently exposed to unnecessary antibiotic therapy, invasive diagnostic procedures, or even surgical exploration [44]. Accurate diagnosis therefore relies heavily on a high index of clinical suspicion combined with targeted imaging—particularly ultrasonography and computed tomography—to identify characteristic amorphous calcifications and surrounding inflammatory changes [14, 45]. Recognition of these imaging patterns is crucial for reframing the condition as a self‐limiting, pain‐dominant inflammatory process rather than a structural or infectious pathology.

#### 3.2.1. Peri‐Hip Calcific Tendinitis

Peri‐hip calcific tendinitis represents one of the most frequently reported nonrotator cuff locations and commonly involves the gluteus medius, gluteus minimus, rectus femoris, or iliopsoas tendons [42]. Patients typically present with acute onset of deep groin, lateral hip, or buttock pain, often accompanied by marked restriction of hip motion, antalgic gait, and significant functional impairment [14, 46]. Additional hip‐specific evidence has further characterized the clinical spectrum of peri‐hip calcific tendinitis. In a case series reported by Park et al., acute calcific tendinitis around the hip most commonly involved the gluteus medius, rectus femoris, and gluteus minimus tendons, with most patients presenting with abrupt inflammatory pain and substantial functional limitation despite favorable outcomes following conservative treatment or image‐guided intervention [47]. Pain may radiate toward the thigh or lower back, further obscuring the diagnosis.

Owing to its symptom distribution, peri‐hip calcific tendinitis is frequently misattributed to lumbar spine pathology, trochanteric bursitis, femoroacetabular impingement, or intra‐articular hip disease [42]. Imaging is therefore central to diagnosis. Plain radiographs and computed tomography typically demonstrate ill‐defined or globular calcifications near tendon insertions, while magnetic resonance imaging often reveals extensive peritendinous edema without primary joint involvement—findings that may otherwise be misinterpreted as infection or neoplasm if calcification is overlooked [46]. Despite the dramatic clinical and imaging presentation, prognosis is generally excellent, with rapid symptom resolution under conservative, pain‐oriented management, underscoring the importance of correct early diagnosis [14].

#### 3.2.2. Achilles Tendon Calcific Tendinitis

Calcific tendinitis of the Achilles tendon is considered relatively uncommon and is widely regarded as diagnostically and therapeutically challenging, largely because it frequently coexists with chronic degenerative changes at the tendon insertion. Maffulli et al. reported that posterior heel pain aggravated by weight‐bearing and mechanical loading represents the most common clinical presentation, a pattern that closely resembles insertional Achilles tendinopathy and enthesopathy [48]. Granqvist and Ackermann further emphasized that concomitant retrocalcaneal bursitis is frequently present, contributing to overlapping clinical features and diagnostic uncertainty [49]. Imaging‐based reviews by Pass et al. and Szaro et al. have similarly highlighted that calcific involvement of the Achilles insertion often cannot be reliably distinguished from degenerative insertional pathology on clinical grounds alone, underscoring the need for careful multimodal assessment in routine practice [50, 51].

From an imaging standpoint, the Achilles tendon should be evaluated within the broader framework of “elementary lesions,” which includes intratendinous calcifications, heterotopic ossifications, tendinopathic thickening, partial tears, and enthesopathic changes [52]. Within this spectrum, accurate differentiation between intratendinous calcifications and ossified lesions is of particular clinical importance. Calcifications are typically composed of amorphous hydroxyapatite deposits and may demonstrate variable echogenicity and posterior acoustic shadowing on ultrasound, often with surrounding inflammatory changes. In contrast, heterotopic ossifications exhibit a more organized structure, frequently with cortical and trabecular patterns, and represent a more mature and less reversible process.

Imaging plays a critical role in differentiating acute calcific inflammation from chronic degenerative calcinosis, as calcific tendinitis represents a biologically active, stage‐dependent process rather than a purely structural abnormality [3]. Discrete calcific deposits within or adjacent to the tendon, particularly when accompanied by surrounding soft‐tissue edema or hypervascularity on ultrasound or MRI, strongly support the diagnosis of an acute inflammatory phase [53, 54].

Intratendinous calcifications, particularly during the inflammatory or resorptive phase, may be effectively managed using minimally invasive approaches such as focused extracorporeal shockwave therapy (fESWT) and ultrasound‐guided interventions, including needling or percutaneous lavage, both of which have demonstrated efficacy in promoting calcification resorption and pain relief in calcific tendinopathy [55]. In contrast, ossified lesions represent a more mature structural transformation and are typically less responsive to these conservative modalities, often necessitating surgical excision when symptomatic. Therefore, accurate lesion characterization is essential to guide appropriate treatment selection and avoid ineffective interventions.

Standardized sonographic protocols have been proposed to systematically assess Achilles tendon pathology, emphasizing evaluation of tendon thickness, fibrillar architecture, vascularity (Doppler signal), and the presence and type of mineralized deposits [56]. Such structured approaches improve diagnostic consistency and facilitate differentiation between active inflammatory lesions and chronic degenerative or ossified changes, thereby supporting a more targeted and stage‐adapted management strategy [53].

From a management perspective, careful integration of pain control, temporary load modification, and gradual rehabilitation is essential. Continued excessive loading during acute inflammatory stages may perpetuate nociceptive signaling and delay recovery, underscoring the importance of stage‐adapted, pain‐informed rehabilitation strategies in this anatomically high‐load region [57, 58].

#### 3.2.3. Knee and Other Lower Limb Sites

Calcific tendinitis involving the quadriceps tendon, patellar tendon, or other peri‐knee structures is distinctly uncommon. Previous reports by Deshmukh et al., Varghese et al., and Barry et al. indicate that the available evidence is largely confined to isolated case reports or small case series, rather than systematic or population‐based cohorts [59–61]. From a clinical standpoint, these peri‐knee manifestations are particularly challenging. Dimmick et al. described that acute calcific periarthritis frequently presents with sudden severe pain, localized swelling, warmth, and marked restriction of joint motion, a pattern that is equally applicable to peri‐knee calcific tendinitis [62]. Similar observations were noted by Ahuja et al., who emphasized that such acute inflammatory presentations often obscure the underlying diagnosis on initial evaluation [63]. Consequently, as highlighted by Beckmann et al., these clinical features closely mimic septic arthritis, crystal‐induced arthropathy, or acute inflammatory synovitis, creating a significant diagnostic dilemma in routine practice [64]. In this context, several authors have noted that clinicians are frequently driven to perform urgent diagnostic investigations—including joint aspiration and advanced imaging—not to establish the diagnosis of calcific tendinitis per se, but primarily to exclude infection or other serious intra‐articular inflammatory conditions, as illustrated in recent peri‐knee case reports [63, 65]. Beyond these peri‐tendinous presentations, a more specific and under‐recognized entity is calcification involving the medial collateral ligament (MCL). Anatomically, a synovial bursa (Voshell’s bursa) is located between the superficial and deep fibers of the MCL, creating a potential space into which calcific material may extend or migrate [66]. During the inflammatory resorptive phase, fragmented and softened calcific deposits may disseminate into this bursal compartment, resulting in calcific bursitis of the MCL. Clinically, this condition typically presents as acute medial knee pain with localized tenderness and functional limitation, often in the absence of trauma and frequently mimicking meniscal or inflammatory intra‐articular pathology [66].

In this diagnostic context, careful identification of peri‐tendinous rather than intra‐articular calcifications becomes central to establishing an accurate, pain‐focused differential diagnosis and avoiding misclassification as primary joint pathology [3, 67]. Ultrasound is particularly valuable in this setting, as it allows precise localization of calcific deposits, assessment of associated bursal involvement, and real‐time evaluation of inflammatory changes. Importantly, in selected symptomatic cases refractory to conservative treatment, ultrasound‐guided percutaneous lavage combined with bursal injection has been shown to provide rapid pain relief and functional improvement, highlighting its role as a targeted, minimally invasive intervention [66]. Consistent with observations at other nonrotator cuff sites, conservative management is generally effective, with clinical improvement and symptom resolution typically paralleling progression through the inflammatory resorptive phase of the disease. Recognition of this self‐limiting pattern is therefore essential, not only to reduce unnecessary invasive procedures or empiric interventions but also to appropriately reassure patients regarding the condition’s favorable natural history.

#### 3.2.4. Distal Upper Extremity

Calcific tendinitis of the forearm, wrist, or hand, although uncommon, often presents with abrupt and striking clinical features, including acute pain, swelling, erythema, and marked functional impairment. Several authors have emphasized that this dramatic inflammatory presentation frequently mimics infectious conditions, most notably cellulitis, flexor tenosynovitis, and septic arthritis, thereby representing a well‐recognized diagnostic pitfall [68, 69]. Imaging‐based case series and reviews further confirm that involvement of small flexor or extensor tendons in the wrist and hand is particularly prone to misinterpretation as infection, especially during the acute resorptive phase, unless characteristic calcific deposits are carefully identified on radiographs or ultrasonography [63, 70].

In this diagnostic context, radiographs and ultrasonography typically reveal small, amorphous calcific deposits within the involved flexor or extensor tendons, often accompanied by disproportionately pronounced surrounding soft‐tissue inflammation [30, 71]. Several authors have noted that recognition of this characteristic imaging–clinical pattern is pivotal, as the condition usually follows a self‐limiting course and responds rapidly to conservative treatment [70]. Accurate and timely diagnosis therefore not only helps avoid unnecessary antibiotic therapy or surgical exploration prompted by presumed infection but also facilitates appropriate pain control and early functional recovery. A summary of the major anatomical sites, clinical features, diagnostic considerations, and mimicking conditions is provided in Table 1.

**TABLE 1:** Types and anatomical distribution of calcific tendinitis across different sites.

Category	Anatomical site	Typical tendons involved	Type of calcification	Clinical features	Diagnostic notes	Treatment implications	Mimicking conditions
Rotator cuff	Shoulder (supraspinatus > infraspinatus, subscapularis)	Rotator cuff tendons	Intratendinous hydroxyapatite deposits	Acute or chronic shoulder pain; severe pain in resorptive phase	Well‐established classifications (Gärtner, Sconfienza); US useful for staging	ESWT, barbotage, conservative therapy; stage‐adapted	Septic bursitis, adhesive capsulitis, cervical radiculopathy, rotator cuff tear
Peri‐hip	Greater trochanter, anterior hip	Gluteus medius/minimus, rectus femoris, iliopsoas	Peritendinous or insertional calcifications	Acute hip pain, limited motion, mimics lumbar or intra‐articular pathology	CT/MRI may show extensive edema; calcification may be subtle	Conservative treatment usually effective	Lumbar radiculopathy, trochanteric bursitis, femoroacetabular impingement, septic arthritis
Achilles tendon	Posterior heel insertion	Achilles tendon	Intratendinous calcification vs ossification	Posterior heel pain, load‐related symptoms	Must differentiate from ossification and enthesopathy	ESWT/needling for calcification; surgery for ossification	Insertional tendinopathy, enthesopathy, retrocalcaneal bursitis, heterotopic ossification
Peri‐knee	Quadriceps tendon, patellar tendon, MCL region	Quadriceps, patellar tendon, medial collateral ligament	Peritendinous or bursal calcifications	Acute knee pain, swelling, mimics septic arthritis	US helpful for localization; may require exclusion of infection	Conservative; US‐guided lavage in selected cases	Septic arthritis, gout/pseudogout, meniscal injury, inflammatory synovitis
Distal upper extremity	Wrist, hand, fingers	Flexor carpi ulnaris, extensor tendons	Small amorphous deposits	Acute inflammatory pain, erythema, mimics infection	Radiographs + US critical to avoid misdiagnosis	Rapid response to conservative therapy	Cellulitis, flexor tenosynovitis, septic arthritis
Migration‐related forms	Bursa, muscle, bone	Secondary to rotator cuff deposits	Extratendinous migrated calcifications	Severe inflammatory pain due to bursitis or soft tissue irritation	US/MRI shows migration pattern	May require bursal injection or targeted intervention	Septic bursitis, soft‐tissue abscess, inflammatory arthropathy

## 4. Diagnostic Strategies

Imaging plays an important role in the diagnosis of calcific tendinitis; however, its clinical value lies not simply in identifying calcific deposits but in interpreting imaging findings in relation to pain presentation and disease stage. This concept is grounded in the well‐established stage‐dependent nature of the disease and the frequent dissociation between imaging appearance and symptom severity [4, 5]. This approach is particularly critical in nonrotator cuff calcific tendinitis, where atypical anatomical locations and acute inflammatory pain frequently lead to diagnostic uncertainty [72].

From a classification perspective, several imaging‐based systems have been proposed to characterize calcific deposits and provide indirect insight into disease stage and biological activity. On plain radiographs, the Gärtner and Heyer classification remains the most widely used, distinguishing well‐defined dense deposits (type I), heterogeneous deposits with partially defined margins (type II), and ill‐defined or translucent deposits (type III), broadly reflecting the transition from formative/resting to resorptive phases [35, 73]. Ultrasound‐based classifications, such as the Sconfienza system, further describe calcifications as hard, fragmented, nodular, or fluid‐like, and, when combined with Doppler signal and posterior acoustic shadowing, allow a more dynamic assessment of inflammatory activity and deposit consistency [36, 74]. These classification frameworks are complementary rather than hierarchical and should be interpreted in conjunction with clinical presentation rather than in isolation.

Plain radiography remains a useful first‐line screening tool for detecting calcific deposits and defining their general distribution [72]. In nonrotator cuff sites, radiographic findings may be subtle or easily overlooked, especially in deep or periarticular regions such as the hip or knee [72]. Moreover, radiographic morphology alone provides limited information regarding biological activity or pain severity and should not guide management decisions in isolation [5, 73].

Ultrasonography represents the most informative modality for a pain‐oriented diagnostic approach across both rotator cuff and nonrotator cuff locations. Beyond its high sensitivity for calcifications, ultrasound allows assessment of deposit consistency, fragmentation, and surrounding inflammatory changes, which have been shown to correlate closely with pain intensity [33]. Doppler imaging can further demonstrate hypervascularity and soft‐tissue edema, features frequently associated with acute inflammatory phases and severe pain that may appear disproportionate to calcification size [53]. Dynamic assessment and real‐time visualization also facilitate diagnostic clarification in anatomically complex nonrotator cuff regions [56].

Magnetic resonance imaging plays a complementary role, particularly for excluding alternative causes of acute pain such as infection, fracture, or inflammatory arthropathy [5]. However, calcific deposits are frequently underestimated or overlooked on MRI, especially when small or fragmented, and MRI findings should therefore be interpreted cautiously in the evaluation of calcific tendinitis [75]. Computed tomography may provide additional value in selected cases, particularly for deep‐seated or anatomically complex regions, where it allows precise localization and differentiation from ossified or tumoral lesions [52, 76, 77].

From a practical standpoint, a simplified, pain‐oriented diagnostic pathway can be proposed. Initial evaluation should integrate clinical presentation—particularly the presence of acute, disproportionate pain—with first‐line imaging using radiography or ultrasound [7, 35]. Ultrasound is preferred when available, as it enables real‐time characterization of deposit morphology and inflammatory activity [36]. Pattern recognition is then essential: well‐defined dense deposits are more consistent with formative or resting phases, whereas ill‐defined, fragmented, or fluid‐like deposits with hypervascularity suggest the resorptive phase and are more likely to be symptomatic [7]. In cases with severe presentation or atypical features, further evaluation should focus on excluding infection, crystal arthropathy, or other intraarticular pathology, often requiring MRI or, less commonly, CT [35]. This stepwise approach emphasizes integration of imaging findings with clinical symptoms and disease stage, facilitating accurate diagnosis while minimizing unnecessary invasive investigations [7].

**FIGURE 2:**
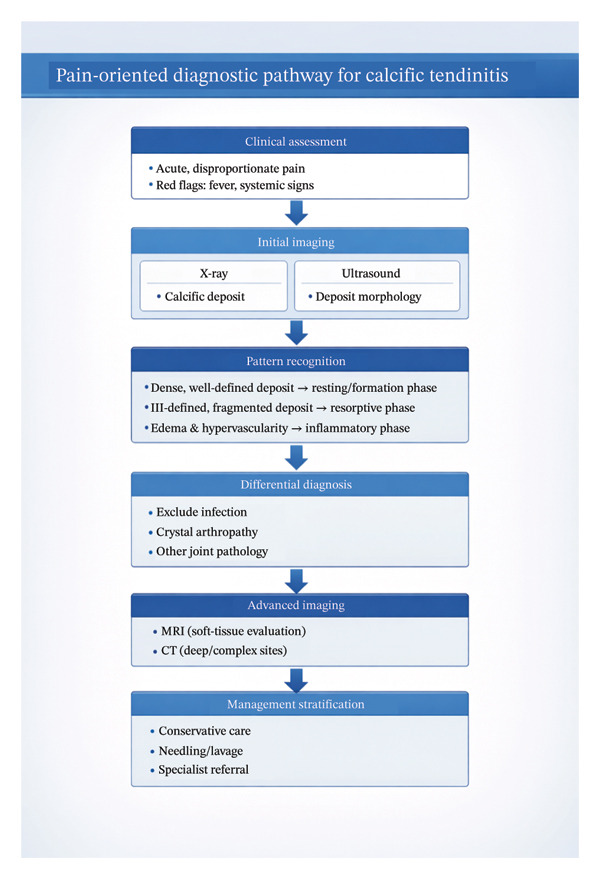
Pain‐oriented diagnostic pathway for calcific tendinitis.

Overall, accurate diagnosis requires integration of imaging findings with clinical symptoms, disease stage, and, when appropriate, laboratory data. Given the often self‐limiting natural course of calcific tendinitis, a pain‐ and stage‐oriented diagnostic strategy is essential for minimizing misdiagnosis and avoiding unnecessary invasive procedures or overtreatment [78]. A simplified pain‐oriented diagnostic algorithm integrating clinical presentation, imaging findings, differential diagnosis, and management stratification is illustrated in Figure 2.

## 5. Treatment Strategies

Management of calcific tendinitis should be individualized and guided primarily by pain severity, functional limitation, and disease stage rather than by the mere presence or size of calcific deposits [5, 8]. Given the often self‐limiting and stage‐dependent nature of the condition, a pain‐oriented, stage‐adapted treatment strategy is essential for optimizing outcomes while avoiding unnecessary interventions [1].

### 5.1. Conservative Therapy

Conservative treatment remains the first‐line approach for the majority of patients and is effective in most cases. Core components include analgesic and nonsteroidal anti‐inflammatory medications, patient education, activity modification, and structured physical therapy programs aimed at maintaining range of motion and restoring tendon–muscle balance [8, 14]. During acute, painful phases—particularly the resorptive stage—treatment priorities should focus on pain control and functional preservation rather than aggressive strengthening [4]. Therapeutic exercise remains the cornerstone of rehabilitation in calcific tendinitis, forming the basis for pain modulation, functional recovery, and restoration of tendon–muscle balance [79].

Among different exercise modalities, eccentric training has been widely recognized as a key component in the management of tendinopathies and is increasingly considered relevant in calcific tendinitis [80]. Eccentric loading is thought to promote tendon remodeling through several mechanisms, including stimulation of collagen synthesis, realignment of tendon fibers, and modulation of neovascularization and nociceptive signaling [80]. In addition, controlled mechanical loading may facilitate the gradual resorption and redistribution of calcific deposits by improving local metabolic activity and reducing intratendinous pressure.

From a practical perspective, exercise programs should be stage‐adapted. During the acute inflammatory phase, low‐load, pain‐limited range‐of‐motion exercises are preferred to maintain joint mobility while avoiding exacerbation of symptoms. As pain subsides, progressive loading strategies can be introduced, with gradual incorporation of eccentric exercises targeting the involved tendon unit. In later stages, combined eccentric–concentric strengthening and neuromuscular control training may further support functional recovery and reduce recurrence risk [81, 82].

Adjunctive physical modalities, including therapeutic ultrasound, may be considered in selected chronic or persistent cases, where they appear to provide modest short‐term symptom relief rather than sustained disease modification [83, 84]. Importantly, conservative strategies should not be regarded solely as an initial step but as an integral component throughout the disease course, particularly in view of the well‐documented tendency toward spontaneous symptom resolution observed across multiple anatomical sites [79].

### 5.2. ESWT

ESWT has gained widespread acceptance as a noninvasive treatment option for patients with calcific tendinitis who remain symptomatic despite initial conservative management. Several randomized trials and systematic reviews have demonstrated that high‐energy fESWT provides more consistent pain relief and functional improvement than low‐energy protocols, particularly in chronic symptomatic cases [85–87]. From a mechanistic perspective, Uhthoff and Loehr and subsequent experimental studies have suggested that the therapeutic effects of ESWT are mediated not only through mechanical fragmentation and promotion of resorption of calcific deposits but also via modulation of nociceptive pathways, neovascularization, and cellular signaling within the tendon microenvironment [88].

Importantly, clinical outcomes following ESWT are strongly influenced by the morphological characteristics of calcific deposits. Soft, fragmented, or fluid‐like calcifications—typically corresponding to the resorptive phase—are generally more responsive to shockwave therapy, likely due to their lower structural cohesion and higher biological activity [89]. In contrast, dense, well‐defined deposits in the formative or resting phases tend to respond less favorably, often requiring higher energy levels or repeated treatment sessions to achieve meaningful clinical improvement [90].

Clinical outcomes following ESWT appear to be stage‐dependent and influenced by deposit morphology, size, and treatment parameters, with better responses typically observed in chronic, well‐defined calcifications compared with acute inflammatory presentations [91, 92]. However, this relationship is not purely structural and should be interpreted in the context of local inflammatory conditions.

In particular, the presence of concomitant subacromial–subdeltoid bursitis or local synovitis may substantially compromise the clinical efficacy of ESWT, even when calcific deposits are clearly visualized. In such scenarios, pain is more likely driven by active inflammatory processes within the bursal or synovial tissues rather than by the calcific burden itself, thereby limiting the symptomatic benefit achievable through shockwave‐induced mechanical fragmentation of deposits.

Emerging clinical evidence indicates that failure to identify and adequately address coexisting synovitis or bursitis is associated with suboptimal outcomes and persistent pain following otherwise technically adequate ESWT [9, 93]. This observation underscores that ESWT is not uniformly effective across all phenotypes of calcific tendinopathy, particularly in cases where inflammatory pathology predominates.

Accordingly, careful patient selection remains critical. In patients presenting with combined tendon and bursal involvement, adjunctive or alternative strategies—such as targeted anti‐inflammatory interventions (e.g., corticosteroid injection or anti‐inflammatory pharmacotherapy)—should be considered to optimize clinical outcomes and avoid treatment failure [93, 94].

On this basis, multiple authors have emphasized that appropriate patient selection is critical to optimize outcomes and avoid overtreatment. ESWT is therefore best positioned as an intermediate therapeutic option, bridging basic conservative care and more invasive interventions such as ultrasound‐guided needling or surgery [95].

### 5.3. Ultrasound‐Guided Needling and Lavage

Ultrasound‐guided needling and lavage (barbotage), often combined with subacromial corticosteroid injection, has been widely used in patients with refractory calcific tendinopathy. While early cohort studies suggested short‐term pain relief and functional improvement (Farín et al., del Cura et al.), these observations have not been consistently confirmed by recent high‐quality randomized controlled trials [96, 97]. In a large double‐blinded, sham‐controlled study, Moosmayer et al. found no clinically relevant superiority of ultrasound‐guided lavage over sham procedures or optimized conservative treatment in terms of long‐term pain and functional outcomes [98, 99]. Similar conclusions were supported by randomized trials and long‐term follow‐up analyses reported by de Witte et al., which failed to demonstrate sustained benefits of barbotage compared with corticosteroid injection alone [33]. From a pain‐management standpoint, ultrasound‐guided procedures should therefore not be regarded as routine treatment escalation [33, 99]. Most authors agree that their use may be justified only in carefully selected patients with persistent, disabling pain, clear imaging–clinical concordance, and inadequate response to comprehensive conservative management [5]. Their potential benefit appears greatest in the resorptive phase, where inflammatory activity is pronounced and short‐term pain modulation, rather than structural eradication of calcification, represents the primary therapeutic target [3].

### 5.4. Surgical Treatment

Surgical intervention should be considered a last‐resort option and limited to patients with persistent symptoms, substantial functional impairment, and failure of exhaustive nonoperative management, given the generally favorable natural history of calcific tendinitis [78]. When surgery is indicated, arthroscopic removal of calcific deposits is widely regarded as the preferred approach, as it permits simultaneous evaluation and treatment of associated pathology such as rotator cuff tears or subacromial impingement [100, 101]. However, several authors have emphasized that surgical debridement does not ensure immediate pain relief, and postoperative pain or stiffness may persist for weeks to months despite satisfactory radiographic and functional outcomes [102, 103]. Therefore, operative treatment should be undertaken cautiously and only after thorough patient counseling regarding the expected recovery course and potential for delayed symptom resolution.

### 5.5. Pain‐Oriented, Stage‐Adapted Treatment Framework

Across all treatment modalities, pain severity and inflammatory activity—rather than calcification size—should guide therapeutic decision‐making. Acute pain episodes are most often linked to the resorptive phase and typically respond well to conservative, symptom‐focused management. Escalation of treatment should proceed stepwise, from noninvasive to minimally invasive and finally to surgical options, with continuous reassessment of pain, function, and disease stage.

## 6. Rehabilitation and Functional Recovery

Rehabilitation is considered a cornerstone of management throughout all stages of calcific tendinitis, contributing to pain relief, functional recovery, and recurrence prevention [104]. Given the stage‐dependent and pain‐dominant nature of the disease, several authors emphasize that rehabilitation should be individualized and dynamically adapted according to symptom severity, inflammatory activity, and anatomical location [9]. During the acute resorptive phase, priorities focus on pain control, tendon protection, and maintenance of joint mobility using gentle, pain‐free range‐of‐motion exercises, while excessive loading should be avoided; as symptoms subside, rehabilitation should progressively shift toward graded loading, strength recovery, and neuromuscular control to support tendon remodeling [104, 105]. For nonrotator cuff calcific tendinitis, site‐specific anatomy and mechanical demands necessitate tailored rehabilitation strategies, as shoulder‐based protocols are not directly transferable.

## 7. Limitations

Several limitations of this review should be acknowledged. First, as a narrative review, this work does not follow a systematic search strategy, and selection bias cannot be fully excluded. Second, evidence regarding nonrotator cuff calcific tendinitis is largely derived from case reports and small case series, which limits the strength of conclusions and precludes the formulation of standardized diagnostic or therapeutic guidelines. Third, pain‐related outcomes and long‐term functional follow‐up—the primary drivers of clinical decision‐making—are inconsistently reported in the existing literature. Future prospective studies with standardized pain and functional outcome measures across different anatomical sites are needed to refine pain‐oriented and stage‐adapted management strategies.

## 8. Conclusion

Calcific tendinitis is best understood as a stage‐dependent, pain‐dominant condition in which symptom severity is driven primarily by inflammatory activity rather than by the size or anatomical location of calcific deposits. Recognition of the dissociation between structural findings and pain is essential for accurate diagnosis and appropriate management. While rotator cuff calcific tendinitis provides a useful reference framework, nonrotator cuff presentations remain under‐recognized and are particularly prone to misdiagnosis because of their acute inflammatory pain patterns. Across anatomical sites, a pain‐oriented, stage‐adapted approach that integrates clinical presentation with targeted imaging offers a more clinically meaningful basis for decision‐making than reliance on imaging morphology alone. Conservative, symptom‐focused treatment combined with progressive, site‐specific rehabilitation should remain the foundation of care, with interventional and surgical options reserved for carefully selected patients with persistent, refractory pain.

**Nomenclature**
CT: Calcific tendinitis
RCCT: Rotator cuff calcific tendinitis
ESWT: Extracorporeal shockwave therapy
fESWT: Focused extracorporeal shockwave therapy
US: Ultrasonography
MRI: Magnetic resonance imaging
CT scan: Computed tomography scan
MCL: Medial collateral ligament
ECM: Extracellular matrix
TNAP: Tissue‐nonspecific alkaline phosphatase
TSPCs/TDPCs: Tendon stem/progenitor cells
VAS: Visual analog scale

## Author Contributions

Jiaqi Li and Xiaodi Zou drafted the manuscript. Tao Jiang and Hui Lu contributed to conceptualization, critical revision, and supervision.

## Funding

The authors have nothing to report.

## Disclosure

All authors approved the final version of the manuscript and agreed to be accountable for all aspects of the work.

## Ethics Statement

This is a narrative review and does not involve human participants or animal experiments.

## Consent

The authors have nothing to report.

## Conflicts of Interest

The authors declare no conflicts of interest.

## Data Availability

Data sharing is not applicable to this article as no datasets were generated or analyzed during the current study.
